# Steroid-responsive Purtscher-like retinopathy associated with macular edema in systemic lupus erythematosus: a case report

**DOI:** 10.1186/s12348-026-00630-w

**Published:** 2026-08-10

**Authors:** Faiz Nurboston Fauzi, Muhammad Firmansjah, Yulia Primitasari

**Affiliations:** 1 Department of Ophthalmology, Faculty of Medicine, Universitas Airlangga, Surabaya, Indonesia; 2 Dr. Soetomo General Academic Hospital, Surabaya, Indonesia

**Keywords:** Purtscher-like retinopathy, Systemic lupus erythematosus, Autoimmune retinopathy, Azathioprine, Case report

## Abstract

**Purpose:**

To report a case of Purtscher-like retinopathy (PLR) associated with macular edema in a young person with systemic lupus erythematosus (SLE) whose vision was restored with a systemic corticosteroid regimen and to highlight the rare and vision-threatening complication of SLE.

**Method:**

Case report.

**Results:**

A 27-year-old female without significant ophthalmological history who had stopped taking azathioprine and methylprednisolone for her SLE presented with sudden painless unilateral vision loss (best-corrected visual acuity [BCVA] RE: 1 m CF, LE: 20/20). Fundus examination revealed bilateral Purtscher-flecken and cotton wool spots, while optical coherence tomography (OCT) identified intra- and subretinal fluid representing macular edema with paracentral acute middle maculopathy (PAMM) on the right eye. The findings led to PLR diagnosis, and the patient received IV methylprednisolone with tapering. Five months after initial encounter, the patient’s vision recovered (BCVA RE: 20/30, LE: 20/20) and OCT showed resolution of the pathology.

**Conclusion:**

While PLR is rarely found in people with SLE, it should be considered as a differential diagnosis in people with SLE presenting with sudden vision loss due to the condition being easy to diagnose clinically and its prompt treatment using corticosteroids may restore the vision loss and resolve the retinal pathology.

## Introduction

Purtscher’s retinopathy is a rare, vision-threatening occlusive microvasculopathy typically associated with severe cranial trauma and manifests as sudden vision loss. The condition is characterized by fundoscopic findings, such as cotton-wool spots, intraretinal hemorrhages, and polygonal areas of retinal whitening known as Purtscher-flecken [1]. Similar findings in the absence of trauma are classified as Purtscher-like retinopathy (PLR) [2].

PLR is a rare diagnosis, and its pathogenesis remains poorly understood. No guideline for PLR has been established, and its management is often empirical, focusing on the treatment of the underlying disease (such as systemic lupus erythematosus, SLE). Immunosuppressants, such as corticosteroids, are the usual choice of therapy [3]. Here, we present our findings of a case of PLR in a person with SLE treated with methylprednisolone.

## Materials and methods

This is a case report conducted in Dr. Soetomo General Academic Hospital on a patient admitted through our emergency department. The patient has given us consent to be followed for study purposes and to have her anonymized data published.

### Case report

In March 2025, a 27-year-old female came to our emergency department with sudden painless vision loss on her right eye that began one week before presentation. She denied any history of ocular trauma, spectacle use, or prior ocular surgery. However, she reported a history of SLE, diagnosed in early 2024, for which she had been prescribed with azathioprine and methylprednisolone. She had discontinued both medications two months prior to the onset of her symptoms.

**Fig. 1:**
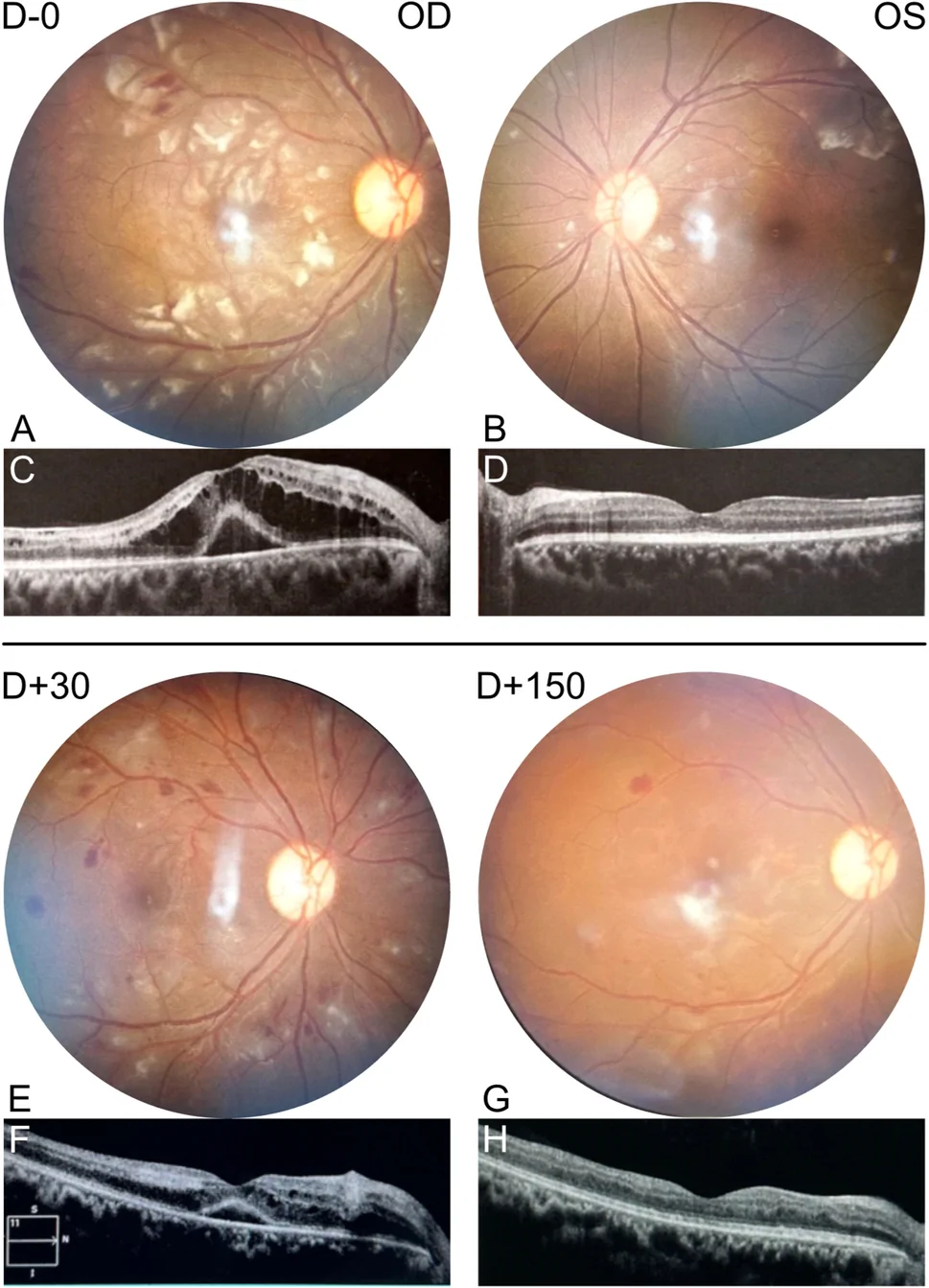
Initial fundoscopy (RE: **A**, LE: **B**) and OCT B-scan (RE: **C**, LE: **D**) of the patient’s retinas, with follow-up of the right retina on one month (fundoscopy: **E**, OCT B-scan: **F**) and five months (fundoscopy: G, OCT B-scan: H) after initial encounter. Note the Purtscher-flecken and cotton-wool spots more prevalent in the right retina during initial encounter, and there was also macular edema in the right eye as seen on OCT

Upon general physical examination, we observed malar rash and alopecia. Visual acuity examination revealed a best-corrected visual acuity (BCVA) of one meter CF RE and 20/20 LE. The results of the physical examination of the eye and the anterior chamber of both eyes were unremarkable, and intraocular pressure was within normal range. Upon fundoscopy, we found multiple polygonal white patches with perivascular clearing (Purtscher-flecken), peripapillary cotton-wool spots, retinal hemorrhage, and optic disc edema on both eyes, revealing classic signs of Purtscher-like retinopathy (Fig. 1A-B). Optical coherence tomography (OCT) revealed intra- and subretinal fluid representing macular edema with paracentral acute middle maculopathy (PAMM) (Fig. 1C-D), while Humphrey visual field testing showed arcuate and double arcuate scotomas. Examination with Ishihara plates also revealed color vision deficiency on the left eye, failing to read 12 out of 28 plates. Upon laboratory examination, mild leukocytosis with elevated erythrocyte sedimentation rate was noted with a highly elevated antinuclear antibody (ANA) level, reaching 400 AU/mL (positive threshold: ≥ 40 AU/mL).

We admitted the patient to our ward and administered IV methylprednisolone 250 mg every 12 h for three days to the patient before switching to PO methylprednisolone for taper-off. The patient was then discharged and followed through our clinic. Upon follow-up at one and five months later, the patient’s visual acuity improved (BCVA RE: 20/30, LE: 20/20 at fifth month of follow-up), her color vision deficiency resolved (all 28 Ishihara plates were read correctly), and fundoscopy (Fig. 1E and G) with OCT (Fig. 1F and H) revealed an improvement from baseline. However, follow-up visual field examination revealed remaining defects.

## Discussion

PLR was first described in a patient with acute pancreatitis [4]. Purtscher’s retinopathy and PLR are rare, with a combined incidence rate of 0.24 cases per million per year [5]. PLR shares features with Purtscher’s retinopathy. However, PLR occurs in the absence of trauma and is often associated with systemic conditions such as SLE. It has been reported that visual disturbances, which may be caused by retinal vasculopathies such as PLR, are rather rare in people with SLE. However, its effect on visual acuity varies and depends on retinal involvement, from no effect on acuity to permanent vision loss [6].

The pathophysiological process underlying PLR remains incompletely understood. While several hypotheses have been discussed, the most widely accepted hypothesis involves microembolization, resulting in precapillary occlusion, which leads to microinfarctions of the retinal nerve fiber layer [2]. These ischemic events manifest as Purtscher-flecken, polygonal areas of retinal whitening with perivascular sparing, a hallmark feature of PLR [6]. OCT B-scans of retina with PLR has demonstrated edema within inner retinal layers with presence of subretinal cystoid spaces and an abrupt transition between affected and unaffected tissue. Potential embolic materials in PLR pathogenesis include fat, air, and leukocyte aggregates, in which the latter might be mediated through autoantigen-autoantibody interaction [3].

While SLE may cause retinal vascular microembolization through the interaction between autoreactive B and T-helper 1 cells causing autoantigen-autoantibody interaction ending with leukoaggregation [7], this interaction is not the sole cause of PLR in people with SLE. Several studies on people with SLE who developed PLR also revealed microvascular changes in the retina; chiefly, a decrease in retinal vessel length, diameter, and density [8, 9]. These changes may be due to autoantigen-autoantibody activity in the retinal microvascular endothelium and may cause microvascular nonperfusion and loss in the retina.

No definitive guideline for the treatment of PLR exists. However, corticosteroids have been used as the treatment of choice. In cases where the use of corticosteroids did not improve the condition, escalation to other immunosuppressants or biologicals is warranted [3]. Such treatment has been noted, for example in a person with PLR whose condition only showed improvement after treatment with eculizumab, a C5 inhibitor [10].

## Conclusion

Purtscher-like retinopathy (PLR) is a rare but significant ocular manifestation associated with autoimmune conditions such as SLE. Although no standardized treatment exists, systemic corticosteroids and immunosuppressive agents remain the mainstay of therapy, with adjunctive interventions applied based on presentation. Prognosis of PLR varies from full recovery to irreversible vision loss and recurrence has been documented, highlighting the importance of early diagnosis and prompt treatment to prevent permanent vision loss.

## Data Availability

No datasets were generated or analysed during the current study.
